# Drink quickly. Mycorrhizal roots deplete water faster from wet soil

**DOI:** 10.1007/s00572-025-01247-y

**Published:** 2025-12-23

**Authors:** David Püschel, Jana Rydlová, Radka Sudová, Jan Jansa, Michael Bitterlich

**Affiliations:** 1 Department of Mycorrhizal Symbioses, Institute of Botany of the Czech Academy of Sciences, Zámek 1, Průhonice, 252 43 Czech Republic; 2 Laboratory of Fungal Biology, Institute of Microbiology of the Czech Academy of Sciences, Vídeňská 1083, Prague 4, 142 00 Czech Republic; 3 Albrecht Daniel Thaer-Institute for Agricultural and Horticultural Sciences, Division Urban Plant Ecophysiology, Humboldt-Universität zu Berlin, Lentzeallee 55/57, 14195 Berlin, Germany

**Keywords:** Arbuscular mycorrhizal fungi, Plant water depletion, Root morphology, Sand–zeolite–soil mixture, *Solanum lycopersicum*

## Abstract

The aim of the experiment was to identify whether mycorrhizal (M) and non-mycorrhizal (NM) plants differed in their rates of water depletion from soil, which was initially very wet but became moderately dry over time. In a greenhouse pot experiment, dwarf-tomato plants (*Solanum lycopersicum* ‘Micro Tom’) were grown in a sterilized substrate. The M plants were inoculated with an isolate of the arbuscular mycorrhizal fungus *Funneliformis mosseae* ‘BEG95’. Moisture meters permanently installed in each pot recorded soil gravimetric water content (GWC) and allowed controlling soil moisture by pot-specific watering. Water depletion was quantified from these GWC data. We found that immediately after water application calculated to establish 17% GWC in each pot, M plants depleted water faster than did their NM counterparts, resulting in less residual substrate water in M as compared to NM pots. The difference in residual substrate GWC remained significant throughout the entire monitoring period of 44 h, with the mean value of the difference being 0.62% of GWC. The plants in the two treatments differed neither in shoot biomass nor leaf area nor root biomass. The M plants, however, had significantly shorter and thicker roots, whereas NM plants had longer, thinner roots. In line with the expectation that thicker roots exhibit higher hydraulic conductivity, we attribute the faster water depletion in M pots to enhanced ability of mycorrhizal roots to exploit wet substrates, where water availability did not constrain uptake. At later stages, both M and NM plants depleted water at similar rates.

**Supplementary Information:**

The online version contains supplementary material available at 10.1007/s00572-025-01247-y.

## Introduction

Considering changes in Earth’s climate and their impacts on precipitation patterns worldwide (Dore 2005; Zhang et al. 2021), the interest of scientists working with arbuscular mycorrhizal fungi (AMF) has become directed toward topics involving water (Abdalla et al. 2023; Augé 2001). The central theme is arguably the assumed higher resistance of mycorrhizal (M) plants to soil water deficiency, be it by means of stomatal regulation (Augé et al. 2015), various molecular and/or genetic mechanisms (Wang et al. 2023), or by altered hydraulic properties of soil (Pauwels et al. 2023). Traditional theory suggests that M plants may simply be able to exploit remaining water more effectively from desiccating soil by means of the extensive networks of AMF hyphae radiating from colonized roots to the soil. These hyphae not only increase the volume of soil within which to seek water (Raven and Edwards 2001), but they also can enter pores physically inaccessible to the roots (Allen 2007; Friese and Allen 1991). Higher resistance of M plants to soil water deficiency may be ascribed in part also to sustained supply of nutrients upon drought as compared to non-mycorrhizal (NM) plants (Bitterlich et al. 2024; Püschel et al. 2021, 2023).

On the other end of the soil moisture spectrum is soil water plentitude, i.e., conditions close to field capacity when most soil pores are water filled. Can AMF, in such scenario, play any significant role in water relations? It has been demonstrated that AMF can slightly yet significantly affect how much water the soil can hold. It is noteworthy that this role can be either positive or negative, depending on soil structure (i.e., the spatial arrangement of soil particles and pores), which can be modified by soil compaction (Püschel et al. 2024). Whether the actual uptake of water from the soil differs between M plants and their NM counterparts has nevertheless remained a neglected question. While it may be an advantageous strategy for the plant to try to exploit as much water as possible at what might be termed the “time of feast,” before the water is drained, evaporated, or taken by competitors, none of the outlined mechanisms relevant for drought is scarcely applicable for wet soils. Could we not therefore assume that both M and NM plants initially exploit water at similar rates and only when water access becomes more difficult do M plants enjoy some advantage?

Here, we report our recent findings from an experiment in which dwarf tomato plants, either M or NM, were grown in pots with sterilized substrate. Each pot was equipped with a permanently installed data logger recording the substrate moisture. Such data not only allowed keeping substrate water content within a certain range by pot-specific watering, but were used also to measure water depletion by the plants in the course of time as substrate water content shifted from high (plentitude of water following the watering) to moderate (partly dried substrate from which the plants already depleted some of the previously provided water). We hypothesized that in wet soil both M and NM plants would deplete water at a similar rate, because it would be easily accessible. Furthermore, we hypothesized that M plants would progressively gain some advantage in access to water as it was depleted and remaining resources became less easily accessible.

## Materials and methods

### Pots with accessories and substrate

The greenhouse experiment was conducted in 2 L pots of our own design, made of thick-walled PVC-U tubes (inner/outer diameter 11.7/12.6 cm) cut to 20 cm length and glued to square plates of the same material. Each pot was equipped with a centrally placed TMS-4 moisture meter (Tomst Ltd., Prague, Czech Republic; www.tomst.com), hereafter referred to as “data logger,” set to record substrate moisture every 15 min. For details on the TMS-4, see Wild et al. (2019). Pot volumes were intentionally reduced to ca. 1.7 L by a 3D-printed funnel-shaped drainage insert installed to avoid waterlogging at the bottom of the pot. Finally, pairs of 3D-printed watering needles were installed close to pot walls, opposing each other. Details on the design and explanation as to the purpose of the drainage insert and watering needles are provided in text and visualization (Fig. S1) in the Supplementary Information.

The sterile substrate was composed of autoclaved (121 °C for 30 min) sand, autoclaved granular zeolite (grain size 1–2.5 mm; Zeopol s.r.o., Břeclav, Czech Republic; www.zeopol.com), and γ-irradiated (> 25 kGy; BIOSTER, Corp., Veverská Bítýška, Czech Republic; www.bioster.eu) soil thoroughly mixed together (9:9:2, v: v:v). The soil component was the same loam as used by and characterized in Pauwels et al. (2023). At the point of filling the pots, the substrate components, which had been weighed in advance for each individual pot, were thoroughly homogenized and at the same time all microbial inoculants were applied (see below). Particular attention was given to achieve the same bulk density in all pots (see Supplementary Information for details), which is highly important for studies addressing soil water availability (Püschel et al. 2024).

### Plants and microbial inoculation

The seeds of dwarf tomato – *Solanum lycopersicum*, cv. ‘Micro Tom’ – were purchased from a local producer (www.permaseminka.cz). After surface sterilization (15 min in 10% sodium hypochlorite) and washing with tap water, the seeds were placed to germinate into a tray with a wet, heat-sterilized sand–zeolite mixture (1:1, v: v). The seeds started to germinate after 4 days. When the radicles were approximately 2 mm long, the seeds were transplanted to experimental pots, one seed per pot, and gently buried in a shallow pit.

Two treatments were established: an M treatment and an NM (or control) treatment. The M treatment was inoculated with 20 mL of suspension of AMF isolate *Funneliformis mosseae* ‘BEG95’, containing ca. 13 000 spores together with mycelium and colonized root fragments. The NM treatment received the same amount of heat-sterilized inoculum (autoclaved twice, 24 h apart, at 121 °C for 30 min) from another culture pot of the same age to equalize the inputs of organic matter in both M and NM treatments. To equalize microbial conditions in the substrate, pots of both M and NM treatments received 15 mL of filtrate from non-sterile soil. Additionally, the NM treatment received 15 mL of microbial filtrate from the original mycorrhizal culture used for inoculum preparation, whereas the M treatment received 15 mL of demineralized water to equalize water inputs. Details on preparation and application of all these microbial inocula are presented in the Supplementary Information.

Each treatment encompassed 33 replicates. The entire experiment thus comprised 66 pots.

### Plant cultivation

The experiment was initiated in the middle of March 2024 and was conducted for 14 weeks until the second half of June in a heated greenhouse equipped with supplementary lighting. LED panels (EuledK 200HS, Euled s.r.o, Prague, Czech Republic) provided a broad spectrum of wavelengths resembling sunlight. Details on light intensities and spectrum of wavelengths, temperature, and humidity inside the greenhouse during the course of the experiment are provided in supplementary Figs. S2 and S3. The positioning of the pots on the tables was randomized at the beginning and in the middle of the experiment.

During the first month, young plants with small/shallow root systems were watered from above as necessary. From mid-April onwards, the watering regime was standardized using moisture data from the data loggers. Each working day, the actual gravimetric water content (GWC) in each pot was measured twice, once in the morning and once in the afternoon. In our system, 1% of GWC corresponded to 21.46 g of water, so the adequate (compensatory) dose of water could be calculated and provided by syringe and the watering needles. This compensated for the difference between the measured and target GWC in each pot. The target GWC was adjusted based on weather but typically ranged around 13% GWC. During weekends, all pots received a unified watering dose, based on the average watering dose for all pots during the preceding week.

The plants were fertilized with a Long Ashton nutrient solution which was applied in doses ranging from 10 mL (at the early stages) up to 60 mL (at the later stages) per pot and application. In total, each plant received 540 mL Long Ashton throughout the experiment, which represented the inputs of 90.7 mg N, 22.1 mg P, 112.3 mg K, 26.1 mg S, and 86.5 mg Ca per pot. The water input provided with fertilization was accounted for in calculations of the actual watering doses.

At the end of May, the first plants started to produce flower buds that were systematically cut thereafter with small scissors to prevent production of fruits and to prolong the vegetative stage of the plants.

### Concept of the water depletion test

Our concept of testing water depletion was based on three assumptions: First, data loggers provide reliable data on the actual moisture. These data loggers utilize the time domain transmissometry (TDT) method to measure soil moisture and provide only “raw data” of the measured signal that need to be converted to GWC values by calibration for a specific soil or substrate. We calibrated the data loggers for our specific substrate and paid attention to achieve similar bulk density during calibration as in the experimental pots. Measurement accuracy of the data loggers is elaborated in the Supplementary Information.

Secondly, we assume that the moisture is rather homogeneously distributed across the entire substrate volume. This is typically not fully achieved with a common watering approach when the pots are watered either at the surface (causing more moisture at the top and drier bottom parts of the profile) or to saucers (resulting in the opposite effect). To greatly improve the homogeneity of water distribution, we provided watering via a pair of needles, as explained in the Supplementary Information. Preliminary tests successfully verified this method.

Finally, we assume that water losses from the pots occur only from plant transpiration. We have empirical evidence that evaporation from plant-free pots of comparable size becomes negligible as soon as the surface layer of the substrate becomes dry (ca. 2–6 g of water evaporated within 24 h). This was the second reason for usage of the watering needles, as this basically minimized moistening of the topmost parts of the soil profile that are then susceptible to immediate evaporation. Moreover, only relatively small amounts of water were provided to the pots before the test stage and these were always held in the substrate (i.e., no free water drained from the pots). In our system, evaporation was thus negligible and drainage was zero. Therefore, we are confident that substrate water depletion can be ascribed solely to uptake by plants.

In morning when the water depletion test was initiated, the average moisture in the pots at 9:00 AM was 13.74% GWC, which was in accordance with our intentions. The plants were neither drought stressed nor exposed to water surplus and thus needed to take up water to maintain their optimal physiological functioning. At 9:30 AM, the pots were watered with pot-specific doses calculated from the latest moisture data. For illustration, the watering doses ranged from 46 to 99 mL per pot with average being ca. 70 mL. After this “final watering,” the moisture should reach the target value of 17% GWC in each pot.

Water provided via the watering needles redistributed itself toward the centrally placed data loggers throughout the substrate occupied by roots. Any differences in the depletion of water in the two treatments were assumed to be detected by the data loggers, and the expected variability of the data (inherent to a methodically complex experimental setup involving biological factors) was counteracted by a large dataset (*n* = 33) for robust statistical comparison of M and NM treatments. As the data loggers are highly accurate in recording relative differences in moisture levels, we could interpret the decrease in GWC over time as depletion of water by the plants. Considering that there were other experimental goals beyond the scope and focus of this study, we could not expose the plants to stronger drought stress and quantify water depletion also in the lowest range of moisture gradient.

### Harvest and data analysis

Plants were harvested 14 weeks after the experiment was established, 1 week after the water uptake was quantified. Plant shoots were cut at the substrate level. Leaf area (LA) was measured using an area meter (LI-3100, LI-COR, USA), then dried at 60 °C to constant weight and quantified as shoot dry weight (SDW). The substrate was shaken off the roots, which were then washed clean with tap water. Subsamples of roots were stained with trypan blue to quantify mycorrhizal colonization by optical microscopy, following the same protocol as previously (Püschel et al. 2020). To describe the morphological characteristics of the roots, the entire root system (excluding the small sample used for colonization) was temporarily stored in 40% ethanol and then spread out in water within a transparent plastic tray. This was scanned at 600 dpi using a flatbed scanner with two-sided illumination (Epson Perfection V800 Photo). The images were analyzed for root morphology using WinRhizo software (Regent Instruments, Quebec City, Canada). As plants had produced a lot of roots, the scanning of the entire root system had to be split into multiple scans (up to 20 scans per plant) to avoid overly dense root distribution on the tray that would have compromised accuracy of the subsequent image analysis. After the scanning, roots were dried at 60 °C to constant weight and quantified as root dry weight (RDW). Total dry weight (TDW) of the plants was calculated as the sum of SDW and RDW. The length of extraradical mycelium was quantified in a sample of the substrate shaken off the roots using a membrane filtration technique (Jakobsen et al. 1992), modified as described previously (Püschel et al. 2007).

The data were analyzed statistically in RStudio Team, version 2025.05.0. (RStudio: Integrated Development Environment for R. RStudio, PBC, Boston, MA, USA; URL. http://www.rstudio.com/), and graphs were generated using the ggplot2 package. Prior to analysis, data were tested for normality and homogeneity of variances. Data for all biometric parameters as well as GWC data passed these tests and the differences between the M and NM treatments were analyzed by *t*-test. The data for water depletion rate in the course of time usually did not meet these criteria and thus were all analyzed by randomization test with 1000 permutations. For the GWC data relevant to the first hours of water depletion, a one-way ANCOVA was conducted to examine the effect of AMF on GWC while controlling for plant biomass as a covariate.

## Results and discussion

The inoculation with AMF successfully established mycorrhizal symbiosis with the inoculated plants, and so the fundamental precondition for testing the hypotheses on water depletion in M and NM systems was achieved. Mycorrhizal structures, particularly hyphae, were found in 57% of root length, and there was a mean of ca. 1600 mm of extraradical hyphae per gram of dry substrate in the M pots. The roots of the NM plants in the control treatment remained uncolonized, with only traces of hyphae present (an average of 27 mm of hyphae per gram of dry substrate). These were most likely old, dead hyphae that were originally present in the soil component of the substrate.

**Fig. 1:**
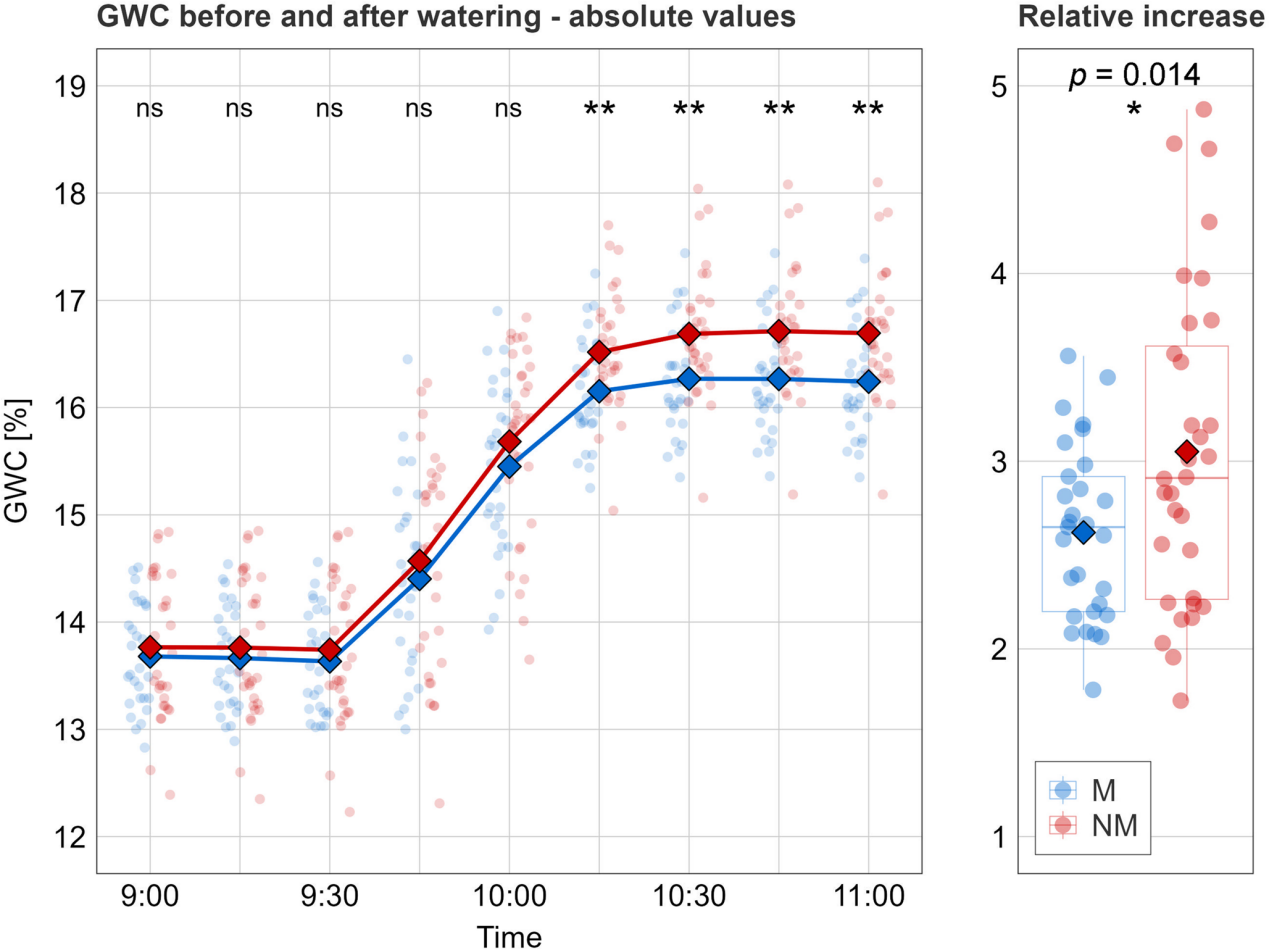
Gravimetric water content (GWC) in the pots as recorded from 9:00 AM to 11:00 AM in 15 min intervals by the data loggers installed in every pot (**A**) and relative increase in GWC that followed the watering (**B**). The watering was applied at 9:30 AM. Mycorrhizal treatment (M) is displayed in blue, whereas non-mycorrhizal control (NM) is red. The dots represent records from individual pots; horizontal variation within each measurement time is determined randomly to separate the dots. Mean values shown as diamond-shape points are connected by lines of respective color to visualize the trends. Nonsignificant differences (ns) in GWC between M and NM pots within each measurement time according to the *t*-test were found up to 10:00 AM, whereas significantly lower (**, *p* < 0.01) GWC values were recorded in M pots thereafter. Significant difference between the two treatments was found also for the relative increase in GWC after the watering (randomization test with 1000 permutations). The results of ANCOVA analysis with plant biomass used as a covariate are shown in Table S1

The final watering was provided at around 9:30 AM to the pots that had similar moisture in both the M and NM treatments (nonsignificant differences between these two groups at that time; Fig. 1). From that point onward, the data loggers recorded a gradual increase in moisture in their surroundings. The curve began to flatten from around 10:15 AM, reaching a plateau approximately 30 min later. From 10:15 AM onwards, a significantly lower GWC was recorded in the M pots compared to the NM pots. Also, the relative data (showing how much the GWC increased from the original to the final moisture) confirmed significant difference between the two treatments (see Fig. 1). To understand what might have caused this difference so shortly after the watering, it must be realized that as soon as the water was applied, it redistributed from the sides of the pots toward the center (where the data loggers were placed) *through the intensively rooted substrate* and thus the plants took part of the water immediately. This also explains why the target moisture (17% GWC) was not fully achieved. During the redistribution, more water was depleted from the soil by M plants than by NM plants, which resulted in significantly lower plateau of moisture reached in M pots than in NM pots. What exact mechanisms are responsible for faster (or more effective) depletion of the water by M plants in these high moisture conditions?

The most obvious explanation would be the size difference between the plants in these treatments. Put simply, bigger plants usually take up more water than do smaller ones. This is not relevant here, however, as we found no significant difference in growth between M and NM plants. In both treatments, the plants grew comparably in terms of all analyzed biometric parameters (SDW, RDW, TDW, and LA) (Fig. S4). Such mycorrhizal growth response, or rather lack of it, is quite consistent with our previous experience with dwarf tomato plants (Püschel et al. 2023). Establishing model plants with comparable biomass in both M and NM treatments makes the findings easier to interpret, as discussed by Püschel et al. (2023). To elaborate on the possible role of plant size in water depletion, we also used one-way ANCOVA to examine the effect of AMF on GWC while plant biomass (SDW, RDW, or TDW) was used as a covariate. Plant biomass turned out to be a significant covariate from 10:00 AM until 11:00 AM, suggesting it to be a significant predictor of GWC (and hence water depletion). Even after adjusting for plant biomass, however, there remained a significant effect of AMF on GWC from 10:15 AM to 11:00 AM (Table S1). This supports our standpoint that the differences in water depletion cannot simply be attributed to a small plant–large plant phenomenon.

Could the explanation be based on the “traditional” concept of largely expanded absorptive apparatus (Raven and Edwards 2001) of M plants compared to NM plants? Extensive hyphal networks can indeed substantially increase the volume of soil from which the nutrients can be exploited and which is inaccessible to the roots alone (Allen 2007; Friese and Allen 1991). Also, some studies have reported significant contribution of AMF to plant water acquisition from root-excluding zones (Kakouridis et al. 2022; Ruth et al. 2011). On the other hand, we are skeptical that hyphal networks per se would provide any meaningful advantage for plant water uptake in our experimental context, i.e., just after watering and when *the roots themselves* were present in the wet soil. In conditions of abundant and larger water-filled pores, which undoubtedly prevailed following the watering, hydraulic continuity between soils and roots persists and does not impose a limitation to root uptake. Considering such high hydraulic conductivity in the wet substrate, we assume that any potential role of AMF hyphae in plant water uptake, be it direct or indirect (Bitterlich et al. 2018a, b; Pauwels et al. 2023), was negligible. We have come to the conclusion that, under these conditions, M plants had no advantage in their *access* to water, but rather in their *capacity* to exploit it.

**Fig. 2:**
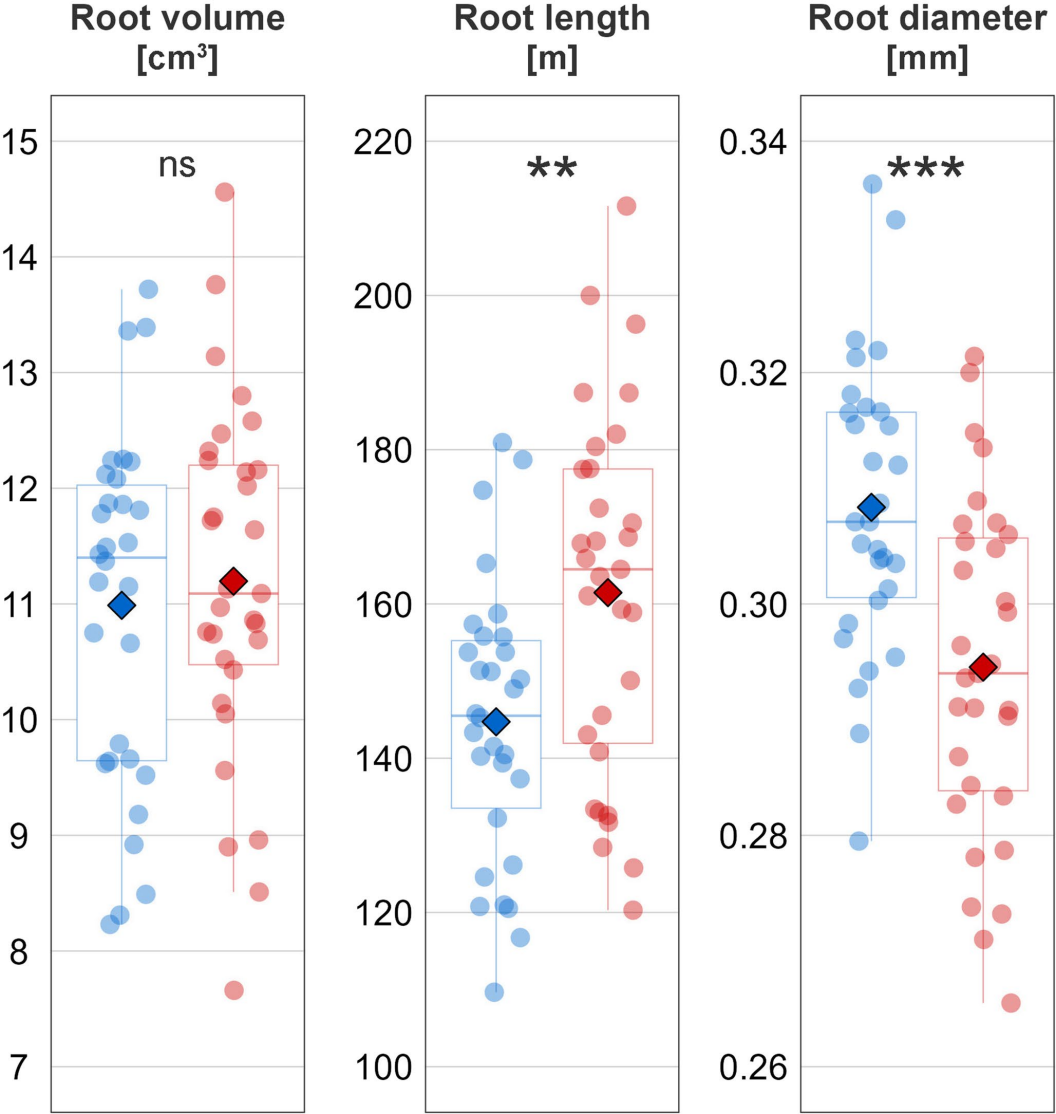
Characteristics of the root system (volume, length, and diameter) of experimental plants. Mycorrhizal (M) plants are shown in blue, whereas control non-mycorrhizal (NM) plants are in red. The round points represent records of individual plants; their horizontal variation within each treatment is determined randomly to separate the points. Center lines of the boxplots indicate the medians, box limits represent the 25th and 75th percentiles, and whiskers extend to 1.5 times the interquartile range. Diamond-shape points indicate treatment means. Significant differences between M and NM plants according to a *t-*test are indicated by asterisks (** 0.001 ≤ *p* < 0.01, *** *p* < 0.001), the symbol ns indicates nonsignificant differences (*p* ≥ 0.05)

We then examined plant root morphology in more detail to find whether the assumption of higher absorptive capacity of M roots is or is not supported. It is known that AMF can change root morphology of their hosts, and this was documented recently also for tomato plants (Shafiq et al. 2023). Generally, in the absence of AMF, plants rely on thin, highly branched roots for their nutrient uptake, whereas the presence of AMF rather promotes thick, unbranched roots (deVries et al. 2021). While RDW of the plants in our study and their overall volume was statistically the same in both inoculation treatments (Fig. S4 and Fig. 2, respectively), M plants had significantly shorter and thicker (in terms of mean diameter) roots as compared to longer and thinner roots of the NM plants (Fig. 2). This observation is in strong agreement with the core conceptual framework established by Bergmann et al. (2020). Interestingly, when the root system of the plants was split into several classes based on their diameters, it was revealed that M plants had significantly fewer roots falling into the thinnest diameter class of < 0.5 mm (Fig. S5). Arguably, the role of these thinnest roots in acquiring resources was partially outsourced to the AMF hyphae. That would agree with deVries et al. (2021) and mean that M plants had a smaller root absorptive apparatus than did NM plants. It is obvious, however, that, under conditions of water plentitude, the absorptive apparatus of the plants did not limit water depletion. The bottleneck was probably rather the “conductive apparatus” (Romero-Munar et al. 2024). Thicker roots might simply conduct more water than do thinner roots, as supported by findings of Kirfel et al. (2017), who concluded that hydraulic properties of small to medium diameter beech roots were mainly determined by root age, thus rendering root diameter a suitable predictor of hydraulic functioning. Indeed, root water uptake models, such as the framework proposed by Couvreur et al. (2012), indicate that, although thicker roots may exhibit higher soil–root radial resistance compared to thinner roots, water uptake under wet conditions is primarily limited by axial root conductance. Indeed, according to Poiseuille’s law, (root) conductivity increases with the fourth power of root radius.

In addition, root colonization by AMF can also elicit physiological reactions that enhance hydraulic conductivity of root systems under wet conditions corresponding to particular gene expression profiles of aquaporins in roots and fungi (Romero-Munar et al. 2024). Even though that study does not inform about root morphological traits and deals with inequality of M and NM plants, it indicates that AMF might also partially contribute to higher root uptake on the physiological level. Nevertheless, further studies are required to determine the extent to which short-term adjustments (aquaporin expression) and long-term adjustments (root morphology) contribute to the increased root water uptake of symbiotic plants after irrigation, as observed in our study. Most studies that have addressed AMF-mediated aquaporin regulation in roots have targeted either drought or salt stress conditions (see Sharma et al. 2021).

The rate at which the plants depleted water from the soil was monitored for 44 h after the watering (Figs. S6 and S7). Climatic data for this specific period are shown in Fig. S3. There were two periods, each lasting several hours, during which M plants depleted slightly yet significantly more water from the substrate than did their NM counterparts: One period occurred in the evening/night of the same day (9–13 h after watering) and the other in the morning of the next day (21–23 h after watering) (Fig. S6). But under further drying of the substrate (i.e., reaching medium moisture), the early advantages of the M plants were compensated for by the NM plants. In fact, the NM plants tended to deplete more water per hour than did the M plants at 27–33 h after watering, which possibly reflected the higher actual GWC in the NM treatment as a consequence of more water being already depleted from the M pots. Arguably, the advantage of M plants in terms of their capacity to transport water was no longer important because the surplus of water had already been depleted and the roots of M and NM plants had become equally effective in acquiring water to satisfy plants’ need in such a scenario. At the same time, mycorrhizal water uptake through hyphae is arguably more important in conditions drier than those reached in this experiment (Allen 2007; Kakouridis et al. 2022). Because in the current study we did not monitor water depletion by M and NM plants to a point of severe drought, we will address that in future experiments by employing a methodology optimized for such specific conditions.

Another aspect worthy of attention is the question of soil hydrophobicity (water repellency). If mycorrhizal rhizosphere is more water repellent, then it may be more difficult for water to pass through such environments. In such case, the lower recorded GWC could be explained by less water coming into the proximity of the sensors. But there is conflicting evidence in this regard across the published studies, with some studies demonstrating a strong correlation (e.g., Rillig et al. 2010; Young et al. 2012) and others denying it (e.g., Feeney et al. 2004; Hallett et al. 2009), especially in controlled environments. An effect of AMF on soil hydrophobicity is thus probably not universally valid and is rather context and/or methods dependent. Nevertheless, this questions certainly is worthy of our consideration in follow-up experiments, where weighing of the pots would help to disentangle this issue. A loss of weight would unequivocally confirm water uptake/transpiration while the opposite would point rather to water remaining outside the reach of the sensors.

## Conclusion

When there was a water surplus shortly after watering, M plants, which were generally comparable in size to their NM counterparts, were able to extract slightly more water from the substrate because their roots were thicker. These thicker roots could thus transfer more water per unit of time than could the comparatively longer, thinner roots of the NM plants. On the second day, when the level of substrate moisture shifted to medium, the advantage of the M plants was no longer demonstrable and the plants in both treatments depleted water at similar rates. Therefore, our hypothesis on the role of AMF in facilitating plant water depletion in progressively drying soils was not confirmed. The positive effect on water depletion by M plants in wet substrate was likely not caused by hyphal networks, but, rather, it was indirect and associated with adaptation of plants’ root systems to mycorrhizal symbiosis. We cannot exclude, however, that the expected role of AMF hyphae on water acquisition would be manifested in even drier soils.

AMF are traditionally viewed as “helpers” that enable plants to survive in adverse conditions, including drought. However, it should be noted that increased water depletion from the soil may also be seen as a double-edged sword. If M plants exploit water faster during a “time of feast,” then, inevitably, the “time of thirst” will come sooner in those specific cases when soil water reserves are not replenished. This may be relevant for plants colonizing extreme habitats or for potted plants. Conversely, in nature, faster water depletion reduces the risk of water draining away, evaporating, or being taken by competing plants. This implies a theoretical double advantage of mycorrhizal plants: Their thicker roots are advantageous when water is plentiful and their thin mycorrhizal hyphae in conditions of soil water scarcity (Kakouridis et al. 2022). The remaining question, however, is whether AMF hyphae can fulfil their role of “roots’ extended arms” and supply *enough* water from a progressively smaller pool of water-filled pores? This simple question calls for further research, which will itself be far from simple.

## Supplementary Information

Below is the link to the electronic supplementary material.

Supplementary Material 1

## Data Availability

No datasets were generated or analysed during the current study.
