# Supplementary material for: Drink quickly. Mycorrhizal roots deplete water faster from wet soil
Source: Mycorrhiza. 2025 Dec 23;36(1):1. doi: 10.1007/s00572-025-01247-y (PMC12727761; doi:10.1007/s00572-025-01247-y)
Supplement: Supplementary file 1 — Supplementary Material 1 [file 572_2025_1247_MOESM1_ESM.pdf]

Drink quickly. Mycorrhizal roots deplete water faster from wet soil

Supplementary Information

Püschel David, Rydlová Jana, Sudová Radka,
Jansa Jan & Bitterlich Michael

Drainage insert

A funnel-form drainage insert of our own design and 3D-printed from Prusament PETG (polyethylene terephthalate glycol copolymer) filament was placed at each pot's bottom (**Fig. S1**) and sealed to the walls with a white sanitary silicone. It was covered with a plastic mesh that held the substrate above the insert (in the upper part of the pot). The insert itself was air-filled. The insert facilitated drainage of excessive water from the pots. Free, superfluous water was led toward the central outlet and dripped freely out of the pot.

Watering needles

These thin, pointed needles of our own design and 3D-printed from Prusament PETG were stuck to the substrate on opposite sides near the pot walls, as shown in **Fig. S1**. The upper end was connected to a short silicone hose to which a 60 mL syringe could be easily attached and the desired amount of water injected into the needle. Its internal channel led the water toward the three outlets spaced 35 mm apart. This system provided more homogeneous water distribution in substrate depth as compared to traditional watering on the pot's surface and allowed for better control over moisture conditions in the pots.

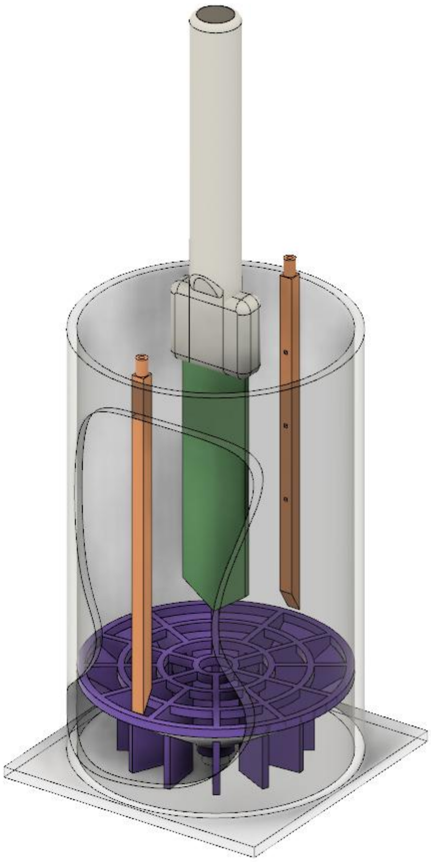

Fig. S1 Visualization of the experimental pots with centrally placed TMS-4 data logger, a pair of watering needles (shown in orange), and a funnel-form drainage insert (violet). The Supplementary Information text explains the purpose of each individual component

Tomst TMS-4 moisture meters (data loggers)

One data logger was inserted into the center of each experimental pot in the orientation as shown in **Fig. S1**. These data loggers were programmed to measure soil moisture every 15 min, which provided optimal temporal resolution. The data loggers were calibrated for the specific potting substrate before being installed in the pots. The calibration employed identical pots, substrate, and plants, as well as correspondingly positioned data loggers, in exactly the same way as in the experiment itself. The pots were watered, after which the moisture content was determined gravimetrically by weighing the pots with consideration for the tare weight and plant biomass. Matching "raw data" from the data loggers were recorded periodically. As the moisture decreased over time due to plant transpiration, a wide range of moisture values was recorded. A calibration curve was constructed from these records and its equation was used to translate the raw values from the data loggers into GWC.

Filling the pots

Sand, zeolite, and soil components of the substrate for each individual pot were weighed in advance and temporarily stored in zip-lock bags. This ensured uniform composition of the substrate in each pot. The sand was first mixed with soil. To this mixture, zeolite and 20 mL of demineralized water were added and thoroughly homogenized. Then 500 mL of substrate were taken, placed into the pot and compressed with 5 free-fall strokes (10 cm height) of a 950 g piston (3D-printed to match pot diameter and filled with sand). Additionally, 250 mL of substrate were taken and put aside. To the rest of the substrate all microbial inoculants (i.e., mycorrhizal inoculum, autoclaved microbial inoculum, and microbial filtrates – as explained in the main text) were added and thoroughly homogenized. This inoculated substrate was then added to the pots in two doses, each compressed with a piston as described above. Finally, the top layer of 250 mL composed of non-inoculated substrate was added to the top; this was a preventive measure taken to minimize the risk of cross-contamination of the pots during the early stage of the experiment when the pots were watered simply on the substrate surface.

Arbuscular mycorrhizal fungal (AMF) cultures and preparation of mycorrhizal inoculum

The isolate *Funneliformis mosseae* 'BEG95' was cultivated in the AMF collection of the Department of Mycorrhizal Symbioses (Institute of Botany, Czech Academy of Sciences, Průhonice, Czech Republic) in sand–zeolite substrate with 10% of soil. The cultures were established with *Zea mays* as the initial host plant and *Desmodium* sp. as the follow-up, long-term host plant. Abundant sporulation of *F. mosseae*, as well as an absence of contamination by other AMF (morphospecies) were confirmed microscopically during a quality check prior to use. The liquid inoculum was prepared by washing ca 1.6 L of substrate from the culture pot through a pair of sieves (200 and 36 µm) and cutting the roots of *Desmodium* to ca 5 mm pieces. Quantity of spores in 1 mL of inoculum suspension was analyzed in three samples, yielding 556, 811, 648 spores per sample. Mean count of spores was thus ca 670 spores. As the inoculation dose comprised 20 mL of suspension, we assume that each pot received ca 13 400 spores of *F. mosseae* 'BEG95'.

Preparation of microbial filtrates

The microbial filtrate from the non-sterile soil was prepared by shaking ca 200 g of non-sterile soil (the same as used in the experiment) with 1.2 L of demineralized water and filtering twice through a 25 µm sieve. A dose of 15 mL was then provided to every pot. The microbial filtrate from the inoculum was prepared by shaking ca 100 g of substrate from the original pot culture with 0.6 L of demineralized water for 30 min and filtering twice through a 25 µm sieve. The dose of 15 mL was then provided to every NM pot.

Data loggers' measurement accuracy and advantages

TMS-4 data loggers show extremely consistent values during repeated measurements of moisture in the same conditions. There is basically zero fluctuation of the recorded values, which enables identification of even the slightest relative changes of water content over time in a given pot. Furthermore, permanently installed data loggers eliminate the issue of soil disturbance that is typically associated with using other moisture meters that require piercing the soil with the probe's rods for every measurement. Finally, data loggers allow comparison of data from all pots at the same time point and at whatever sampling time point throughout the experiment.

Moisture conditions before and during the test stage

It can be questioned whether the plants in the two treatments compared were cultivated in the same moisture prior to the test itself. In other words, can we exclude any undesirable differences in moisture between M and NM pots that could potentially affect plant physiology and thus skew the water depletion test? To address these concerns, we have performed a few confirmation tests (a *t*-test was used in all these cases). First, we analyzed the data loggers' data and calculated the mean daily GWC in each pot for each of the 6 days that preceded the water depletion test. The results provided evidence that the moisture conditions were indeed equal in both treatments every day (**Fig. S8**). Also, in the morning when the water depletion test started (i.e., before the final watering was applied), there was nonsignificant difference in GWC (**Fig. 1**). Further, the calculated pot-specific water inputs provided to each pot – not only during the final watering but also during the days that preceded the water depletion test – did not differ significantly between M and NM pots (data not shown). Finally, we plotted the average daily watering dose against total plant biomass and found that in both M and NM treatments plant biomass correlated positively with the watering applied (**Fig. S9**). This was fully expected, as large plants consume more water in wet soil or, more precisely, need to be watered more, to get to a certain GWC. This was a necessary correlation documenting that the pot-specific watering was performed as intended. From these confirmation tests, we conclude that the experiment was set well and there was no bias between the treatments either in moisture conditions or in water inputs that could have skewed the results of the water depletion test.

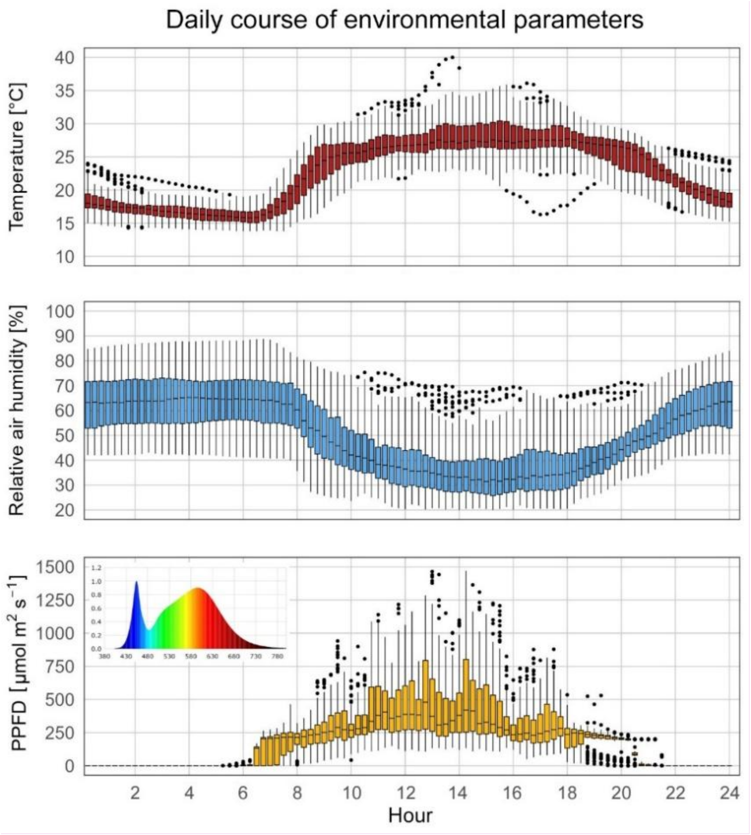

Fig. S2 Visualization of environmental parameters – temperature, relative air humidity, and photosynthetic photon flux density (PPFD) – recorded in 15 min intervals by the data logger installed inside the greenhouse. The visualized data were collected throughout the whole course of the experiment (n = 83). The inserted histogram of wavelengths emitted by the LED panels of supplementary lights was provided by a certified laboratory for light measurement, METROLUX, Ltd., Prague, Czech Republic (www.metrolux.cz)

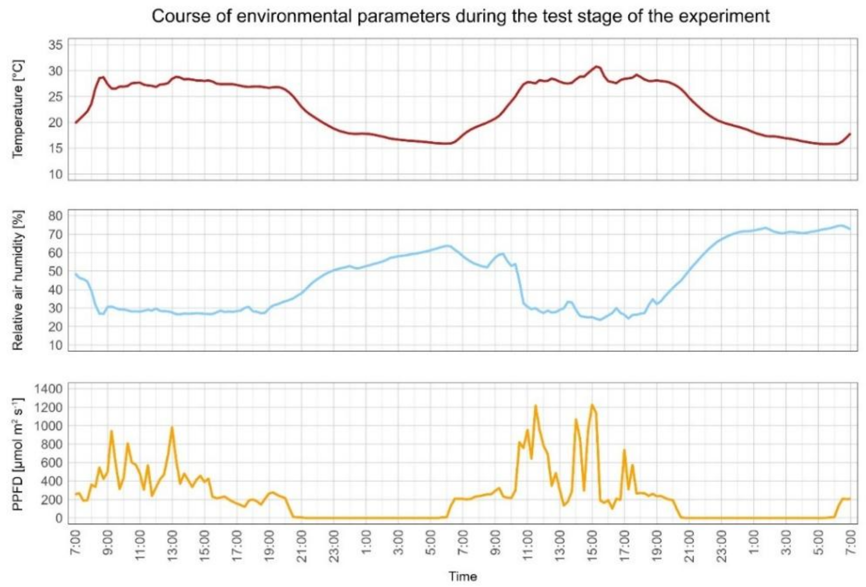

Fig. S3 Visualization of environmental parameters – temperature, relative air humidity, and photosynthetic photon flux density (PPFD) during the test stage of the experiment when water uptake by the plants was quantified

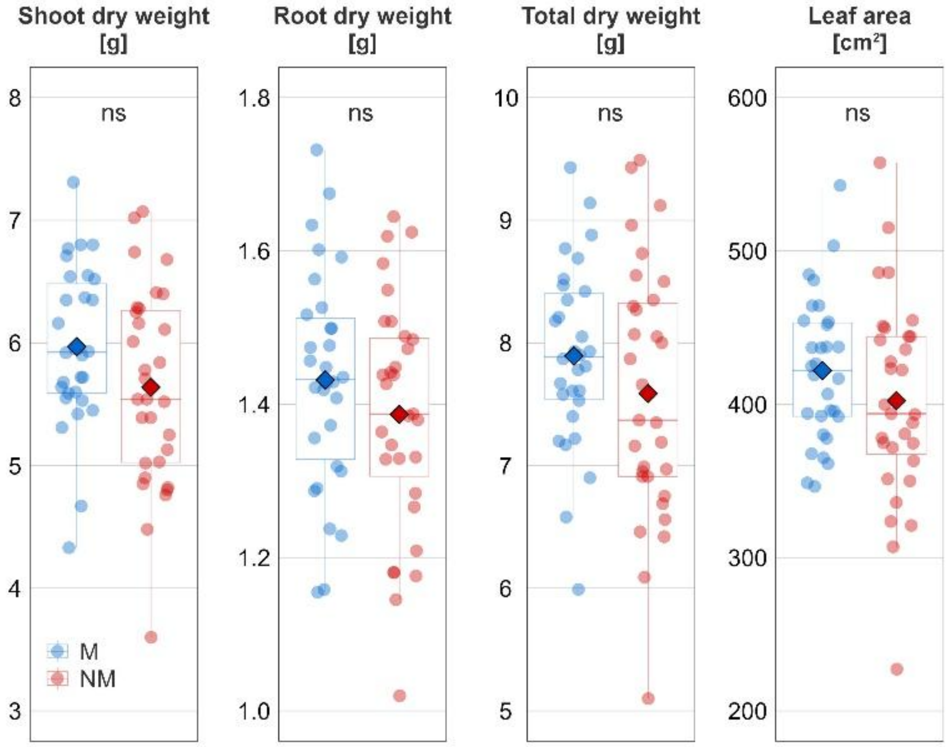

Fig. S4 Biometric parameters of experimental plants – shoot dry weight, root dry weight, total dry weight, and leaf area of experimental plants. Mycorrhizal (M) plants are shown in blue color, whereas control non-mycorrhizal (NM) plants are in red. The dots represent records of individual plants; their horizontal variation within each treatment is determined randomly to separate the dots. Center lines of the boxplots indicate the medians, box limits represent the 25th and 75th percentiles, and whiskers extend to 1.5 times the interquartile range. Diamond-shape points indicate treatment means. The symbol "ns" indicates nonsignificant differences between M and NM plants according to a *t*-test

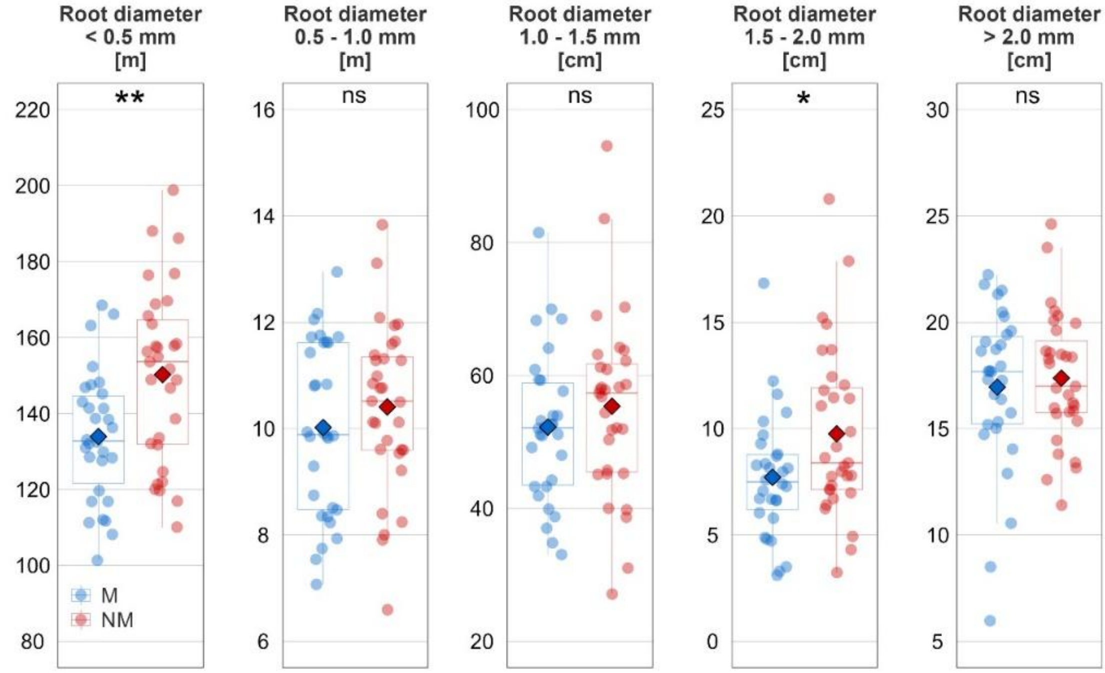

Fig. S5 Length of roots sorted into five classes based on their diameters. Note that the first two categories are shown in meters, whereas the other three categories are in centimeters. Mycorrhizal (M) plants are shown in blue color, whereas control non-mycorrhizal (NM) plants are in red. The dots represent records of individual plants; their horizontal variation within each treatment is determined randomly to separate the dots. Center lines of the boxplots indicate the medians, box limits represent the 25th and 75th percentiles, and whiskers extend to 1.5 times the interquartile range. Diamond-shape points indicate treatment means. Significant differences between M and NM plants according to a *t*-test are indicated by asterisks (* $0.01 \le p < 0.05$, ** $p < 0.01$); the symbol “ns” indicates nonsignificant difference

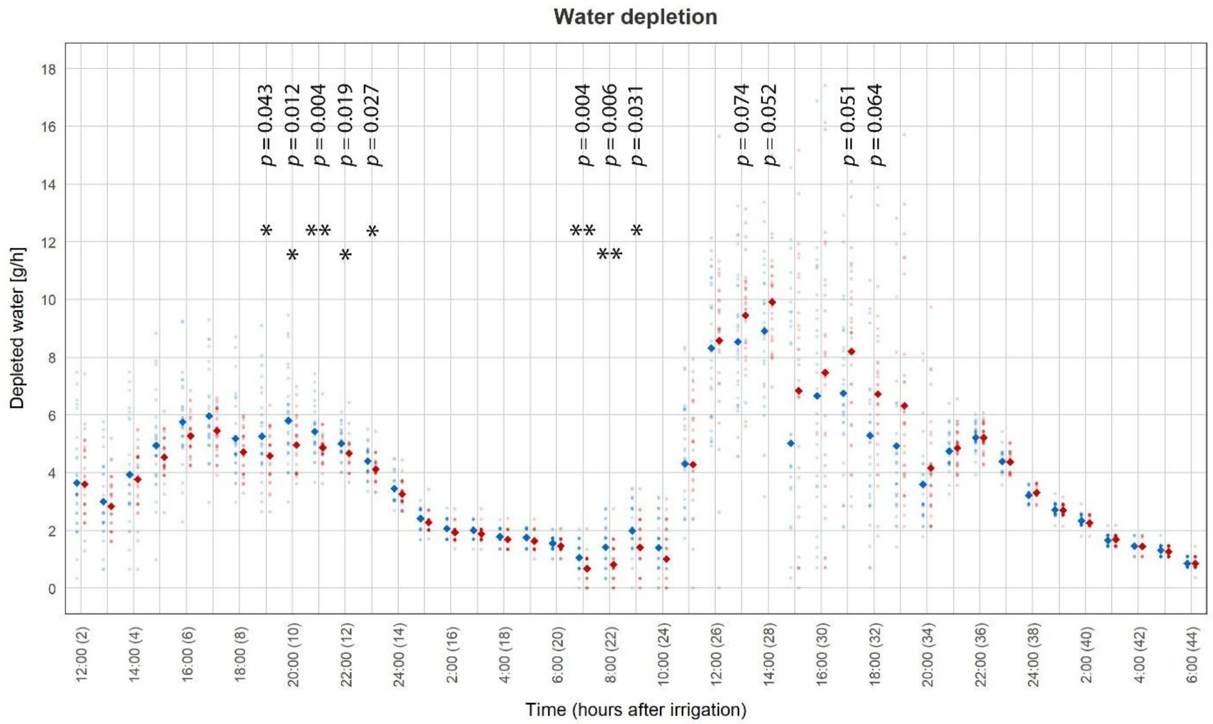

Fig. S6 Rate of water depletion by the plants during 44 h following the watering. Mycorrhizal treatment (M) is displayed in blue color, whereas non-mycorrhizal control (NM) is red. The dots represent records from individual pots. Mean values are shown as diamond-shape points. Significant differences between the two treatments according to randomization test with 1000 permutations are indicated by asterisks (* $0.01 \le p < 0.05$, ** $p < 0.01$) and shown together with *p*-values (which are also shown for close-to-significant instances). Increased depletion rates during daytime coincided with higher temperatures, lower humidity, and, of course, higher irradiance as shown in Fig. S3 of the supplementary material

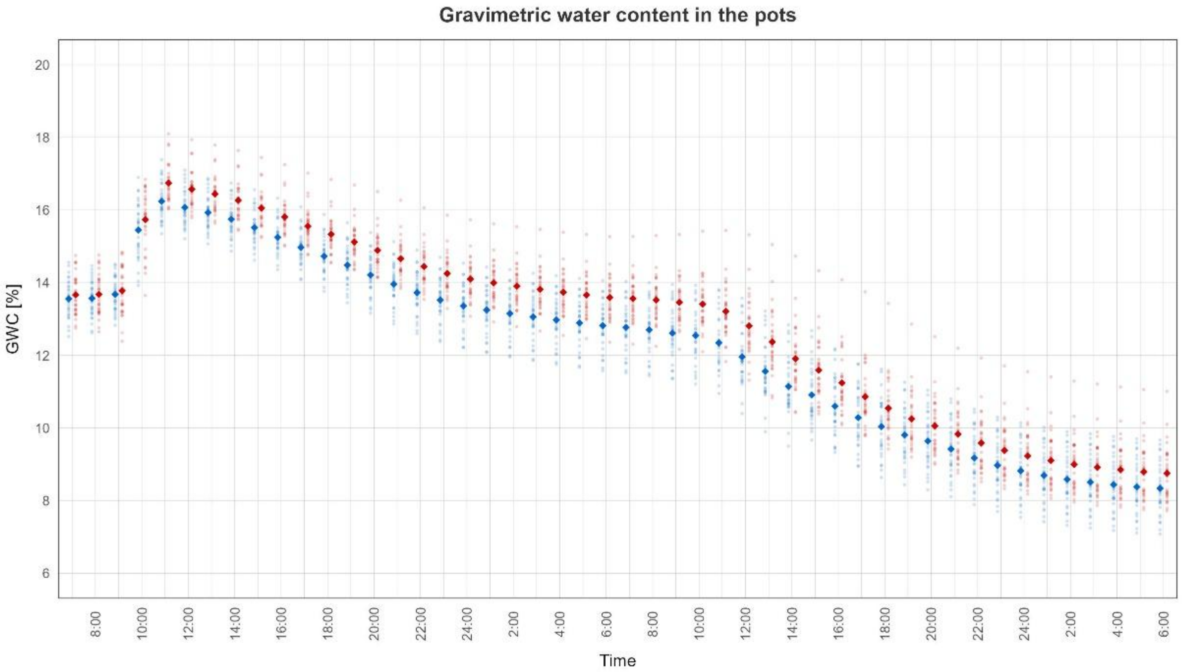

Fig. S7 Gravimetric water content (GWC) in the pots throughout the entire test period as measured in 60 min intervals by the data loggers installed in every pot. Mycorrhizal treatment (M) is displayed in blue color, whereas non-mycorrhizal control (NM) is red. Dots represent records from individual pots; mean values are shown as diamond-shape points. Significantly lower GWC ($p < 0.01$ according to the *t*-test) was consistently recorded in M pots in each measurement time starting at 11:00

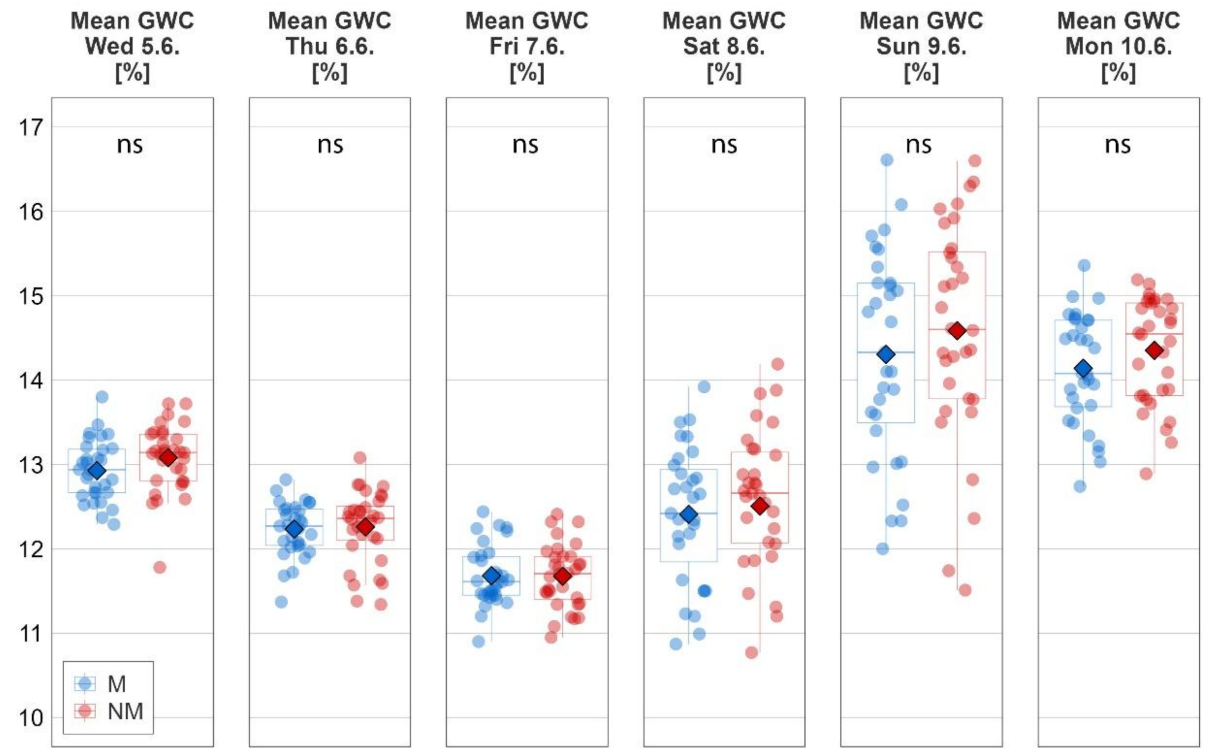

Fig. S8 Daily mean gravimetric water content (GWC) in the pots as recorded every 15 min for 24 h and shown for 6 days (Wednesday to Monday) that preceded the test stage of the experiment. Mycorrhizal (M) pots are shown in blue color, whereas control non-mycorrhizal (NM) pots are in red. The dots representing individual pots are means of 96 daily records from the data logger, their horizontal variation within each treatment is determined randomly to separate the dots. Center lines of the boxplots indicate the medians, box limits represent the 25th and 75th percentiles, and whiskers extend to 1.5 times the interquartile range. Diamond-shape points indicate treatment means. In neither case was there a significant difference between M and NM pots according to a *t*-test, as indicated by the "ns" symbol. Higher variability of data on Saturday and particularly on Sunday are caused by uniform watering, whereas pot-specific watering applied during working days (as described in the main text) resulted in more standardized moisture in the pots

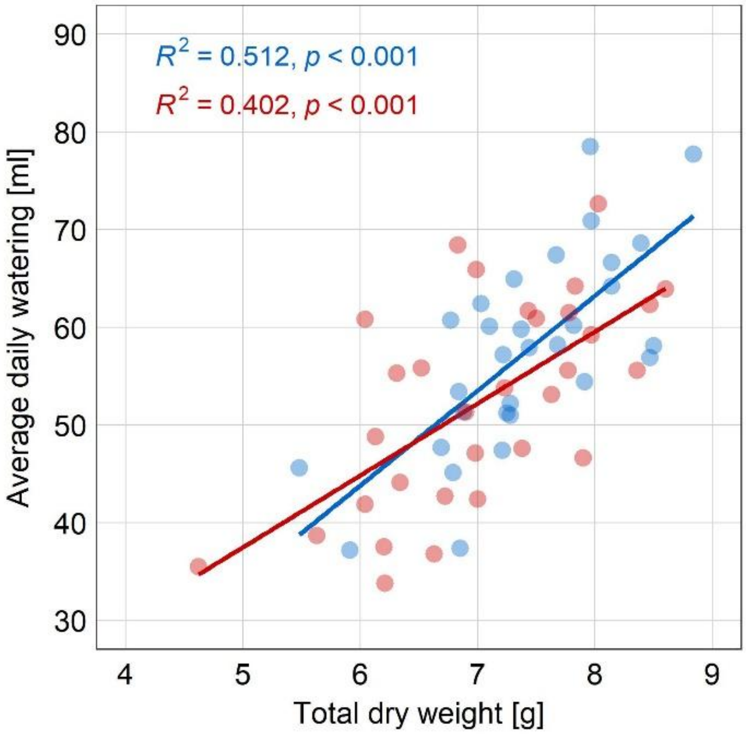

Fig. S9 Correlation of daily watering dose (average across 4 days of pot-specific watering) and total dry weight of the plants. Mycorrhizal (M) plants are shown in blue color, whereas control non-mycorrhizal (NM) plants are in red. Linear regression lines are shown together with R^2 and p values. A linear model was used to test average daily watering as a function of total dry weight of the plants, presence of arbuscular mycorrhizal fungi, and their interaction. The interaction was found to be nonsignificant (p = 0.3485), meaning that the slopes of the two separate regression lines are not significantly different

Time	Covariate SDW	AMF	Covariate RDW	AMF	Covariate TDW	AMF
9:00	ns	ns	ns	ns	ns	ns
9:15	ns	ns	ns	ns	ns	ns
9:30	ns	ns	ns	ns	ns	ns
9:45	ns	ns	ns	ns	ns	ns
10:00	*	ns	ns	ns	*	ns
10:15	***	**	*	**	***	**
10:30	***	**	*	**	***	**
10:45	***	**	*	***	***	**
11:00	***	**	*	***	***	**

Table S1 Effect of plant biomass (either shoot dry weight – SDW, root dry weight – RDW, or total dry weight – TDW) used as a covariate and of the presence of arbuscular mycorrhizal fungi (AMF) used as the main explanatory factor on water content in the experimental pots within a timespan from 9 AM to 11 AM. The assumption of homogeneity of regression slopes was tested by including the interaction term (AMF × respective biomass); it turned out not significant, indicating that the relationship between the biomass and GWC did not differ significantly between the two groups and ANCOVA analysis can be used. The asterisks indicate significant effects of the covariate or AMF (* $0.01 \leq p < 0.05$, ** $0.001 \leq p < 0.01$, *** $p < 0.001$) while ns denotes that the effect was not significant. The effect of a covariate turned out to be nonsignificant for the relative increase of GWC that followed the watering (Fig 1B)
